# The effect of macrophages and their exosomes in ischemic heart disease

**DOI:** 10.3389/fimmu.2024.1402468

**Published:** 2024-05-10

**Authors:** Minrui Wang, Chunhong Li, Yuchang Liu, Yuanyuan Jin, Yang Yu, Xiaoqiu Tan, Chunxiang Zhang

**Affiliations:** ^1^ Department of Physiology, School of Basic Medical Sciences, Southwest Medical University, Luzhou, Sichuan, China; ^2^ Department of Pharmaceutical Sciences, School of Pharmacy, Southwest Medical University, Luzhou, Sichuan, China; ^3^ The Key Laboratory of Medical Electrophysiology of the Ministry of Education, Southwest Medical University, Luzhou, Sichuan, China; ^4^ Department of Cardiology, the Affiliated Hospital of Southwest Medical University, Luzhou, Sichuan, China

**Keywords:** ischemic heart disease, immune cells, macrophage, extracellular vesicles, exosomes

## Abstract

Ischemic heart disease (IHD) is a leading cause of disability and death worldwide, with immune regulation playing a crucial role in its pathogenesis. Various immune cells are involved, and as one of the key immune cells residing in the heart, macrophages play an indispensable role in the inflammatory and reparative processes during cardiac ischemia. Exosomes, extracellular vesicles containing lipids, nucleic acids, proteins, and other bioactive molecules, have emerged as important mediators in the regulatory functions of macrophages and hold promise as a novel therapeutic target for IHD. This review summarizes the regulatory mechanisms of different subsets of macrophages and their secreted exosomes during cardiac ischemia over the past five years. It also discusses the current status of clinical research utilizing macrophages and their exosomes, as well as strategies to enhance their therapeutic efficacy through biotechnology. The aim is to provide valuable insights for the treatment of IHD.

## 1 Introduction

Cardiovascular disease (CVD) is a leading cause of global mortality, with over 18.5 million deaths attributable to CVD in 2019, with ischemic heart disease (IHD) accounting for half of all CVD-related deaths worldwide (1). IHD is characterized by reduced blood flow to the heart, leading to an imbalance between myocardial oxygen supply and demand. Ischemia of the myocardium can progress to ischemia-reperfusion arrhythmias, myocardial infarction (MI), and even heart failure (2).

Due to its complex pathophysiological mechanisms, understanding the specific mechanisms involved in IHD occurrence can contribute to the development of more effective treatment methods aimed at improving patient survival rates (3–6).

The underlying mechanisms of most CVDs involve innate and acquired immune responses. Among them, inflammation is one of the important complications following IHD, such as MI or reperfusion injury, and the inflammatory cascade response plays an important role in myocardial tissue injury, repair, and remodeling, and mastery of the cell-specific signaling mechanisms that mediate the inflammatory response is essential for the treatment of MI (7–11).

Immune cells are involved in microenvironmental changes following the development of IHD, and macrophages are among the most abundant immune cells in the heart (12, 13).

In healthy conditions, cardiac-resident macrophages constitute 6–8% of non-myocardial cells in adult mice (14). After myocardial ischemia, cardiac macrophages undergo marked changes in phenotype and function and are capable of massive expansion through their proliferation and recruitment of monocytes, a behavior implicated in both the injurious and reparative responses of the heart (7, 15, 16). This is primarily evidenced by the rapid apoptosis of resident macrophages within 2 hours and their near depletion within 24 hours following MI (17). Meanwhile, disruption of cardiac homeostasis leads to recruitment and differentiation of Ly6C^hi^ monocytes from the bloodstream into macrophages, replacing resident macrophages and persisting long-term (18–20). Upon ischemic insult, circulating monocytes swiftly transition from a rolling to a flowing state via activation of chemokine ligand 2 (CCL2)/monocyte chemoattractant protein (MCP)-1 signaling (21). This prompts their infiltration into the infarcted area, forming a reservoir of monocytes. Stimulated by factors such as colony-stimulating factor and granulocyte growth factor within macrophage colonies, these monocytes differentiate into mature macrophages, thus constituting the primary source of cardiac macrophages (22).

While resident macrophages are scarce in number, recent research indicates their ability to proliferate following cardiac injury, thereby influencing the subsequent recruitment of monocytes. Following cardiac injury, resident macrophages produce inflammatory and chemotactic factors responsible for clearing and degrading apoptotic cardiomyocytes, impacting cardiac conduction (23–25). These resident macrophages are typically divided into CCR2^-^ and CCR2^+^ subsets, each with distinct mechanisms and functions (26). Tissue-resident CCR2^+^ macrophages promote monocyte recruitment through a MYD88-dependent mechanism, leading to MCP release and monocyte mobilization, while CCR2^-^ macrophages inhibit monocyte recruitment (27).

Nevertheless, both in terms of quantity and impact, resident cardiac macrophages are not as influential as circulating monocytes, which play a predominant role in the ischemic heart (28). Circulating monocytes differentiate into M1 type early post-MI and transition to M2 type later (29). Classically activated M1 macrophages primarily engage in phagocytosis, MHC II antigen presentation, and reactive oxygen species production (30–32), whereas M2 macrophages stimulate extracellular matrix production, cell proliferation, and angiogenesis, facilitating tissue remodeling and repair (33–35). In conclusion, the dynamic interplay between resident cardiac macrophages and circulating monocytes plays a pivotal role in the response to MI and subsequent cardiac repair processes. Further investigation into the precise molecular pathways governing macrophage behavior in the injured heart holds promise for the development of targeted strategies to enhance cardiac healing and functional recovery.

Exosomes are extracellular vesicles with a diameter of 40–160 nm that are secreted by cells and serve as important vehicles for paracrine signaling (36). Exosomes play pivotal roles in various macrophage-mediated effects, serving crucial functions in cellular processes. Their biogenesis involves distinct stages, commencing with cellular internalization, where the cell membrane engulfs extracellular material to form vesicles. Subsequent fusion with endosomal compartments generates early endosomes, which mature into intraluminal vesicles (ILVs) within late endosome multivesicular bodies (MVBs). Ultimately, MVBs merge with the cell membrane, releasing ILVs as exosomes (37, 38). Exosomes play a significant role in intracellular and intercellular communication by selectively delivering cargoes such as nucleic acids, proteins, and lipids to target cells and organs. They contribute to important processes including angiogenesis, ventricular remodeling, and immune response regulation following cardiac ischemia (39). The therapeutic benefits of exosomes have been validated in various animal and disease models (40–42). Moreover, the unique physiological properties of exosomes, including their circulatory stability, biocompatibility, low immunogenicity, and low toxicity, make them an excellent carrier for targeted drug delivery, which has been applied in a range of diseases such as cancer and inflammation (43). As typical secretory cells, macrophages are capable of secreting various molecular signaling substances and releasing different types of exosomes in the ischemic microenvironment (44–46). Based on this, this review summarizes the research progress in the regulatory mechanisms and therapeutic effects of different phenotypic macrophages and their exosomes in IHD over the past five years. It evaluates their specific biological functions and provides guidance for the utilization of macrophage-derived exosomes in low-risk, highly targeted therapy for IHD.

## 2 The role of macrophages in IHD

Due to the non-regenerative nature of adult cardiac cells, prolonged myocardial ischemia often leads to MI, resulting in irreversible loss of cardiomyocytes and even left ventricular remodeling and progressive heart failure (47). Therefore, protecting cardiomyocytes and preventing ventricular remodeling and heart failure are important strategies for treating IHD.

**Figure 1:**
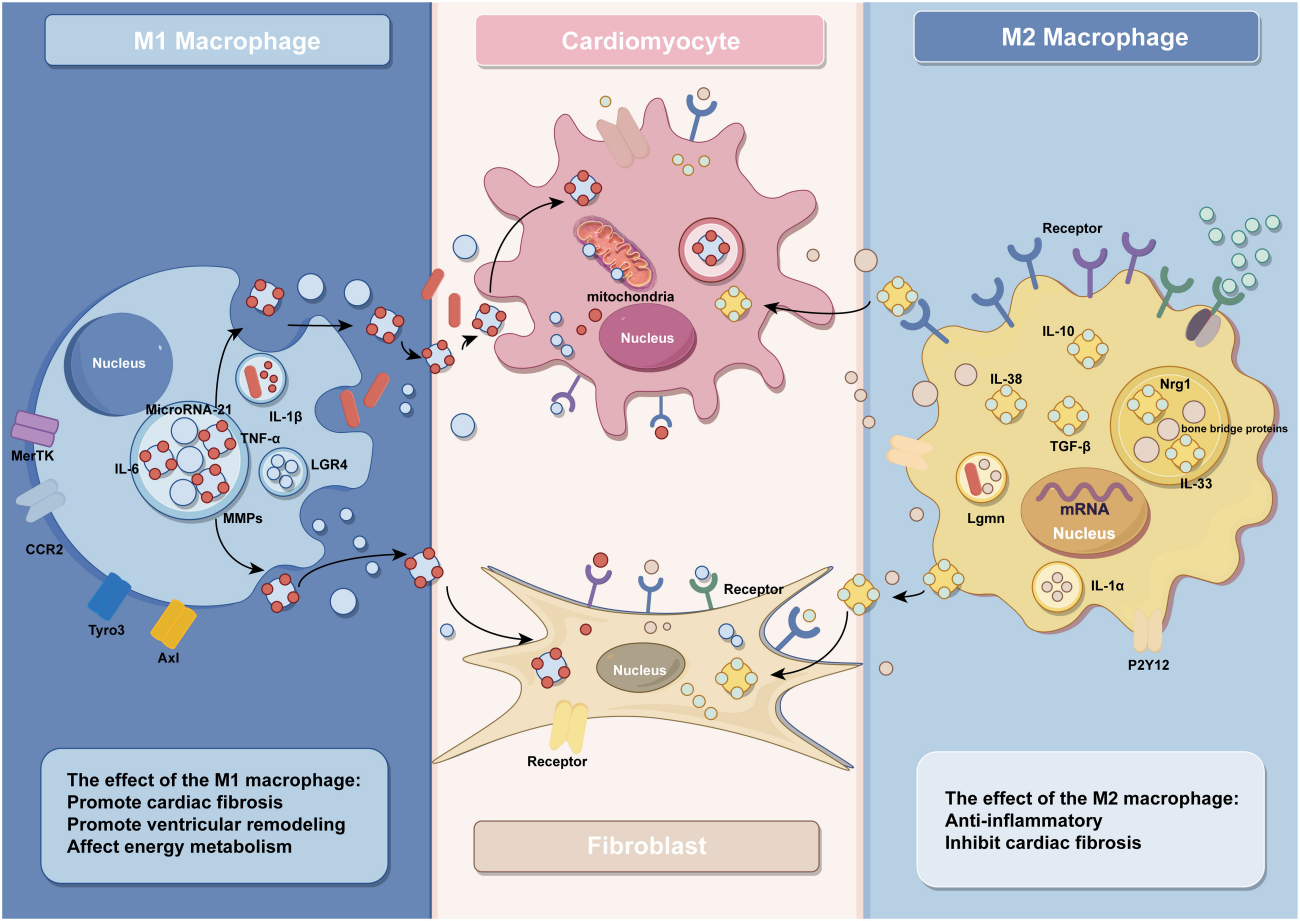
The effect of macrophages and their exosomes in IHD (by Figdraw).

Studies have shown that ventricular remodeling is closely associated with dysregulated immune responses, and monocytes and macrophages are the key effector cells of the immune system (48). During disease onset, a significant accumulation of blood-derived monocytes occurs at the ischemic site of the heart, which subsequently differentiate into macrophages to participate in the immune response (3). In the early phase of MI, the necrosis of a large number of cardiomyocytes triggers an intense inflammatory response, and the number of M1 macrophages will peak at 3–4 days after MI, participating in the removal of damaged cells and phagocytosis of extracellular matrix debris during this inflammatory phase (49). In the following days, the inflammatory phase gradually transitions into the reparative phase, where macrophages shift towards an M2 phenotype. M2 macrophages become predominant from day 5 post-infarction and secrete anti-inflammatory and pro-fibrotic cytokines to facilitate the repair of injured myocardium. It is noteworthy that this transition depends on the timely suppression of the inflammatory response. Prolonged activation of M1 macrophages can lead to extensive cardiomyocyte death, degradation of extracellular matrix, expansion of the infarct area, and adverse ventricular remodeling, ultimately resulting in heart failure (4, 49–54) (**Figure 1**).

### 2.1 The role of M1 macrophages in IHD

#### 2.1.1 M1 macrophages are involved in the pro-inflammatory and fibrotic responses after MI

M1 macrophages in IHD are mainly involved in the inflammatory response of post-infarction myocardial tissue and myocardial tissue fibrosis, thus aggravating cardiac injury (55). As a result of myocardial necrosis, the integrity of ECs and their barrier function are impaired, promoting the release of danger-associated molecular patterns, further activating intercellular crosstalk signaling and releasing a large amount of pro-inflammatory mediators, facilitating polarization of macrophages towards the M1 phenotype (56). Activated M1 macrophages release a significant amount of inflammatory cytokines and growth factors such as tumor necrosis factor-α (TNF-α), interleukin-1 (IL-1), chemokines, etc., which further contribute to the promotion of inflammation and fibrotic responses. For example, angiotensin II (AngII) AT1 receptors are involved in the development of myocardial fibrosis through stimulation of the TNF-α/NF-κB/CD44-triggered κ-signaling pathway (57, 58). Studies have shown that chemokines are key linking factors between myocardial inflammation and fibrosis, such that CC chemokine ligand 2(CCL2) can exert fibrotic effects by recruiting and activating M1 macrophages expressing its receptor CCR2 (59). In addition, C-X-C chemokine receptor 4 (CXCR4) is a vital regulator of macrophage-mediated immune responses, and CXCR4 significantly enhances the expression of chemokine (C-X-C) motif ligand (CXCL3), thereby promoting myofibroblast (MF) differentiation (60). In addition to various cytokines, some microRNAs contained in macrophages regulate the inflammatory and fibrotic responses after MI. For instance, studies conducted by Deepak Ramanujam et al. have demonstrated that microRNA-21 (miR-21) is not only the most abundant microRNA in cardiac macrophages but also a key factor contributing to myocardial tissue fibrosis. M1 macrophages secrete miR-21 in a paracrine manner, targeting cardiac fibroblasts (CFs) and promoting their transition from a quiescent state to MFs (61). After MI, there are significant changes in the protein expression levels within resident macrophages in the heart. Among them, the synthesis and degradation of matrix metalloproteinases play multiple roles in the process of ventricular remodeling. Seven days after infarction, the expression of Mmp14 (MT1-MMP) in macrophages significantly increases. Specific deletion of Mmp14 in mice can significantly alleviate post-MI cardiac dysfunction, reduce fibrosis, and protect the microvascular network in the heart (16). A series of pro-inflammatory reactions induced by M1 macrophages promotes the occurrence of cardiac fibrosis, further leading to impaired cardiac contraction and ejection function, exacerbating the development of heart failure. Therefore, early and rapid intervention and modulation of the secretion of relevant cytokines and gene expression can effectively prevent the progression of IHD.

#### 2.1.2 Involvement of M1 macrophages in ventricular remodeling

Patients with IHD undergoing reperfusion therapy still face the challenges of left ventricular remodeling and heart failure after MI (62). The polarization of macrophages is regulated by multiple proteins, and a reduction in the expression of protective proteins after MI promotes the polarization of macrophages towards the M1 phenotype, exacerbating the inflammatory response and leading to adverse ventricular remodeling. For example, decreased expression of V-set and immunoglobulin domain containing 4(VSIG4), a protein that protects against cardiac injury after ischemia in myocardial tissue, further activates TLR4/NF-κB and accelerates macrophage polarization toward M1 macrophages, which leads to increased apoptosis of cardiomyocytes and aggravates cardiac injury after reperfusion (63). On the other hand, ischemia in the myocardium dramatically increases the expression levels of various proteins that promote M1-type polarization, further exacerbating the development of ventricular remodeling. Among them, Dectin-1, a class of proteins that regulates macrophage differentiation, is highly expressed in the early phase of cardiac ischemia-reperfusion (I/R), and its elevated expression leads to the increased polarization of macrophages toward M1 type and promotes infiltration of Ly-6C^+^ monocytes and neutrophils, leading to further myocardial injury and ventricular remodeling (64). In addition to this, macrophages in the early stages of MI highly express Lgr4, which leads to inflammatory macrophage activation by promoting cAMP response element binding protein(CREB) mediated c-Fos, Fosl1 and FosB trans activation, further leading to reduced cardiac function, increased myocardial infarct size and poor ventricular remodeling (65). As with Dectin-1 and Lgr4, increased expression of grass bacillus proteinogenic-converting enzyme 9 (PCSK9) in myocardial tissue after acute MI promotes poor myocardial repair by the polarization of M1 macrophages (66–68).

Following MI, macrophages undergo polarization toward M1 phenotype, thereby instigating the inflammatory cascade. Furthermore, certain cytokines released by macrophages impede their differentiation into M2 subtype. Consequently, this cytokine-mediated hindrance compromises the myocardial tissue repair mechanism. For example, the increased expression of YAP and TAZ after MI further increases the secretion of IL-6 by M1 macrophages by interacting with the histone deacetylase 3 (HDAC3)-nuclear receptor co-blocker 1 (NCoR1) blocking complex, thereby decreasing arginase-I (Arg1) expression, further impeding the repair response (69). Timely regulation of protein expression and maintenance of macrophage homeostasis would contribute to modulating the healing process following ischemic injury. For example, research by Wang et al. demonstrated that inhibition of purinergic receptor 2Y12 (P2Y12) in macrophages reduced inflammation and improved reperfusion arrhythmias in a rat I/R model, which served as cardioprotection (70). Relevant references are also presented in **Table 1** for easy visualization (**Table 1**).

**Table 1:** Effect of M1 macrophage on IHD.

Cell Source	Target pathway	Effect	Reference
Mouse macrophages	TNF-α/NF-κB/CD44	Promote cardiac fibrosis	(57)
Mouse macrophages	MCP-1/CCL2		(59)
Mouse macrophages	CXCR4		(60)
Mouse bone marrow-derived macrophages	microRNA-21		(61)
Mouse macrophages	MT1-MMP/TGFβ1		(16)
Rat macrophages	VSIG4/TLR4/NF-κB	Promote ventricular remodeling	(63)
Mouse macrophages	Dectin-1		(64)
Mouse macrophages	Lgr4/CREB/c-Fos、Fosl1、FosB		(65)
THP-1-derived macrophages and human primary macrophages	PCSK9/TLR4/NF-κB		(66, 68)
Mouse macrophages	YAP、TAZ/HDAC3- NCoR1/IL-6/Arg-1		(69)
Rat macrophages	P2Y12		(70)

### 2.2 The Role of M2 macrophages in IHD

Following early MI, cardiac-resident macrophages become depleted, which promotes adverse cardiac remodeling in the peri-infarct area and severely impairs cardiac function. However, activated M2 macrophages, similar to cardiac-resident macrophages, have the capability to promote tissue repair and regulate the homeostasis of the myocardial microenvironment, thereby exerting crucial cardioprotective functions (**Table 2**) (25, 78).

**Table 2:** Effect of M2 macrophage on IHD.

Cell type	Target pathway	Effect	Reference
human primary macrophages	IL-38/JNK/AP1	Anti-inflammatory	(71)
Mouse macrophages	IL-10/hyaluronidase-3/hyaluronic acid	Anti-inflammatory	(34)
Mouse macrophages	TGF-β/lncRNA ATB	Anti-inflammatory	(72)
Mouse macrophages	Nucleolin/Notch3、STAT6	Anti-inflammatory	(73)
Mouse macrophages	Notch signaling	Anti-inflammatory	(74)
Mouse macrophages	ALK4	Anti-inflammatory	(75)
Mouse macrophages	Legumain	Anti-inflammatory	(23)
Mouse macrophages	IL-1α;osteopontin	Inhibit cardiac fibrosis	(76)
Mouse macrophages	Nrg1/ErbB	Inhibit cardiac fibrosis	(77)

#### 2.2.1 Involvement of M2 macrophages in the anti-inflammatory response after MI

M2 macrophages safeguard the ischemic heart by secreting anti-inflammatory factors, including IL-10, IL-38, and transforming growth factor-β (TGF-β), thereby serving as potent anti-inflammatory agents. Among them, IL-10 can promote the polarization of macrophages to M2 macrophages and improve the cardiac microenvironment through M2 macrophage-dependent hyaluronidase-3/hyaluronic acid degradation mechanism to further subdue the inflammatory response and promote myocardial tissue healing (34). IL-38, a newly discovered member of the IL-1 family, de-activates the c-jun N-terminal kinase/activator protein 1 (JNK/AP1) pathway by binding to interleukin one receptor helper-like protein one and increases IL-36 production, regulates dendritic cell-induced cardiac regulatory T cells, thereby modulating macrophage polarization and improving myocardial post-infarction ventricular remodeling (71). In contrast to IL-10 and IL-38, a recent study has shown that transforming growth factor-β (TGF-β) can reduce cardiomyocyte inflammatory response, oxidative stress, and cell apoptosis through activation of the long non-coding RNA ATB, providing multiple avenues to alleviate cardiac I/R injury (72).

In addition to cytokines, a variety of proteins play important roles in the anti-inflammatory process of M2 macrophages. Legumain, a gene specifically expressed in M2 macrophages, is involved in the post-myocardial infarction (MI) inflammatory response by upregulating IL-10 and TGF-β, while downregulating IL-1β, TNF-α, and IL-6 (23). Similar cardioprotective proteins include nucleolin, which was previously found to significantly attenuate myocardial I/R injury by promoting myocardial angiogenesis and reducing cardiomyocyte apoptosis by Tang et al (79). Furthermore, their subsequent investigation revealed a substantial decrease in nucleolin expression during the early stage of MI, followed by an increase during the later stage. This upregulation of nucleolin, facilitated by the key regulatory factors notch homolog 3 (Notch3) and signal transducer and activator of transcription 6 (STAT6), promotes M2 macrophage polarization, thus contributing to the anti-inflammatory response (80). It is worth noting that the role of the Notch signaling pathway in ischemic myocardium is dual-edged. Activation of the Notch pathway has been shown to suppress ventricular remodeling in MI rats, but overexpression of Notch signaling may have fibrotic effects on cardiac fibrosis (73, 81, 82). Besides, another study has demonstrated that blockade of the Notch signaling pathway promotes M2 polarization of cardiac macrophages and improves cardiac function by inhibiting imbalanced fibrotic remodeling after MI (74).

Promoting the differentiation of macrophages to M2 macrophages by regulating the expression of specific genes in mice has emerged as a target for modulating the anti-inflammatory response. A study by Yuli Yang et al. found that inhibition of ALK4 gene expression in mice significantly inhibited the secretion of inflammatory factors by M1 macrophages while inducing a phenotypic switch from pro-inflammatory M1 macrophages to anti-inflammatory M2 macrophages and ultimately promoting cardiac repair after myocardial injury (75).

#### 2.2.2 Role of M2 macrophages on myocardial fibrosis after MI

M2 macrophages exhibit anti-inflammatory and tissue repair properties by producing high levels of anti-inflammatory cytokines and promoting fibroblast progenitor cell proliferation and differentiation, playing a crucial role in CFs-mediated myocardial repair (50). Analogous to macrophages, these CFs adopt a pro-inflammatory phenotype soon after MI, after which they differentiate into MFs, which secrete anti-inflammatory factors and extracellular matrix proteins to repair and stabilize cardiac tissue (83). M2 macrophages can secrete IL-1α and osteopontin to activate CFs and promote their transformation into MFs, thereby forming more supportive fibrous tissue at the infarct site and repairing the vulnerable ventricular wall of the infarcted heart (76). In addition to promoting CF activation, M2 macrophages inhibit CF senescence and apoptosis by activating the neuroglial protein 1 (Nrg1)/epidermal growth factor receptor (ErbB) pathway (77).

As the exploration of macrophage functions progresses, the classification of M1/M2 appears simplistic and broad. Mosser and Edwards proposed a classification system for macrophage function, categorizing macrophages into classical activation, regulatory, and repair subtypes (84). Further *in vitro* experiments have subdivided M2 macrophages into four subgroups, including M2a, M2b, M2c, and M2d (85). Among them, M2a macrophages express high levels of fibrogenic factors, contributing to repair during early stages of injury, while M2b macrophages are defined as regulatory cells with potent immunomodulatory and anti-inflammatory effects (85, 86). *In vitro* experiments by Yue et al. demonstrated that M2a macrophages significantly promoted the proliferation and migration of CFs, the expression of fibrosis-associated proteins, and the differentiation to MFs, whereas M2b macrophages had the exact opposite effect on CFs, with a significant antifibrotic effect (86, 87). Subsequently, they evaluated the effects of M2b macrophage transplantation using a rat I/R model, confirming that M2b macrophages can reduce cardiac fibrosis, improve heart function significantly by inhibiting the mitogen-activated protein kinase (MAPK) signaling pathway, and decreasing the activation of platelet-derived growth factor receptors (PDGFRs) in CFs (88).

In addition to the common regulation of cardiac fibrosis through modulating CFs, M2b macrophages can also regulate the fibrotic process by affecting the lymphatic system. Lymphatic vessels in the heart drain interstitial fluid to maintain cardiac homeostasis, and previous studies have shown that exogenous vascular endothelial growth factor C (VEGFC) can stimulate lymphangiogenesis in the heart, thereby alleviating myocardial edema and fibrosis after MI (89). Recent research has revealed that transplantation of M2b macrophages upregulates the expression of VEGFC and vascular endothelial growth factor receptor 3 (VEGFR3) in the hearts of I/R rats, promoting lymphangiogenesis to reduce myocardial fibrosis and improve cardiac function (90). Taken together, this series of studies demonstrates the potential value of macrophage therapy in heart disease. Further investigations are needed to explore the complexity of macrophage subtypes and differentiation, as well as the interplay between different signaling pathways.

### 2.3 Macrophage phenotype switching intimately linked to metabolic responses Resource Identification Initiative

In IHD, the phenotypic polarization and metabolic changes of macrophages recruited into circulation and residing in tissues can disrupt the M1/M2 homeostasis, thereby impacting the balance of cardiac inflammatory effects and determining disease regression and prognosis (37, 38). The polarization and activation of macrophages are closely associated with metabolic reprogramming, which is manifested as a bias in energy utilization, thereby altering their inflammatory phenotype (91). Macrophages primarily modulate their inflammatory phenotype through four energy cycles, including: 1) glycolysis, 2) oxidative phosphorylation (OXPHOS), 3) tricarboxylic acid cycle (TCA), and 4) fatty acid oxidation (91, 92). Under normal physiological conditions, macrophages are intimately integrated to tissue and organismal metabolism (93). M1 macrophages rely predominantly on glycolysis, accelerating glucose transport by upregulating the glucose transporter protein GLUT1 to meet the demands of rapid ATP production, whereas M2 macrophages utilize fatty acids as a fuel source for TCA and subsequent OXPHOS (94, 95).

In IHD, changes in cardiac metabolism may disrupt M1/M2 homeostasis, which in turn affects the cardiac inflammatory response and further influences disease regression and prognosis. Studies have shown that ischemia leads to an increased dependence of cells on glycolysis, while hypoxia induces pro-inflammatory gene expression and metabolic reprogramming towards glycolysis (96, 97). Following myocardial tissue injury, macrophages will activate a series of receptor tyrosine kinases such as Tyro3, Axl, and MerTK, which mediate the clearance of apoptotic cells and regulate the production of inflammatory cytokines (98). Among these, cross-signaling between AX1 and TLR4 transduces to glycolytic metabolism and pro-inflammatory IL-1β secretion, leading to an increased inflammatory response within the myocardium, unfavorable ventricular remodeling and impaired contractile function (99). Glycolytic metabolism promotes macrophage-induced fibrosis, whereas inhibition of glycolysis facilitates the restoration of macrophage energy metabolism from glycolysis to normal OXPHOS pathway under a normoxic state, further blocking M1 polarization and thus improving the condition of ischemic cardiomyopathy (100–102). This undoubtedly provides excellent targets for targeted therapies to inhibit inflammation, for example, Zhao et al. used salvianolic acid B to inhibit mammalian target of rapamycin 1 (mtorc1)-induced glycolysis, which reduced myocardial M1 macrophages and increased M2 macrophages in mice at 3 days after I/R and reduced collagen deposition and improved cardiac dysfunction at 7 days after I/R (103). Notably, glycolysis does not have only negative effects. Early endogenous glycolytic reprogramming after MI can promote the transcription of reparative genes by promoting histone demethylation in monocytes, thereby improving cardiac function after MI (104).

Metabolic shifting between glycolysis and mitochondrial OXPHOS is an important mechanism for the transition of macrophages to reparative phenotype, and a timely transition of M1 macrophages to M2 macrophages would contribute to myocardial repair (105). On the first day after MI, macrophages polarize towards the M1 phenotype, exhibiting distinct pro-inflammatory and extracellular matrix degradation characteristics. However, by the third day post-MI, macrophage proliferation and phagocytic capacity increase, accompanied by upregulation of genes associated with mitochondrial function and OXPHOS, indicating metabolic reprogramming (106). In diseased states, this metabolic transition is influenced by multiple factors. For instance, comprehensive metabolomic analysis has indicated that the TCA cycle may be interrupted during the inflammatory process, resulting in selective accumulation of intermediates including succinate, which further impacts oxidative phosphorylation and thus the metabolic switch of macrophages. Additionally, the further oxidation of succinate can drive the generation of a large amount of reactive oxygen species (ROS), exacerbating oxidative damage (100, 101, 107, 108). Recent studies have shown that activated macrophages can produce itaconate to inhibit succinate oxidation and regulate M2 macrophage polarization, indicating that macrophages possess certain metabolic regulation capabilities (109). Moreover, alterations in the cardiac microenvironment exert a profound impact on macrophage metabolism, while the activity of macrophages reciprocally influences the heart. For instance, IL-33 can be released by various cells following necrosis. It not only induces macrophage reprogramming, leading to uncoupling of the mitochondrial respiratory chain and increased production of the mitochondrial-derived metabolite itaconate, thus promoting the resolution of inflammation and initiation of tissue damage repair; but also activates the JAK/STAT signaling pathway to induce M2 macrophage polarization, thereby impeding the progression of cardiac fibrosis and improving cardiac systolic and diastolic function (110, 111). Indeed, Liu et al. demonstrated that *in vitro* induced M2 macrophages could be transplanted into hearts of heart failure mice models, confirmed that M2 macrophages can transfer mitochondria to damaged cardiomyocytes, which promote cell survival under stress conditions and alleviate cardiac fibrosis and cardiomyocyte apoptosis (112).

Currently, there have been relevant studies on treating diseases by regulating the polarization state and functions of macrophages at the metabolic level, showing promising therapeutic benefits (95). For example, Chen et al. demonstrated that supplementing with ω-alkynyl arachidonic acid can inhibit glycolysis and promote M2 macrophage polarization, leading to reduced infarct size, prevention of the development of left ventricular dysfunction, and improved clinical outcomes in a mouse model of MI (113). Further investigation will contribute to unraveling the intricate interplay between macrophage energy metabolism and the cardiac microenvironment and deepen our understanding of this dynamic change, advancing the development of relevant therapeutic strategies.

### 2.4 Macrophage phenotype switching intimately linked to microenvironment

Macrophage polarization plays a pivotal role in host defense and tissue repair, with its regulation extending beyond metabolic cues to encompass various microenvironmental factors such as cytokines, cell receptors, and microRNAs. Cytokines, notably, are key determinants in the transition from M1 to M2 macrophage phenotypes (114). Exemplifying this, IL-4 orchestrates the shift from M1 to M2 via JAK1/STAT6 signaling (115). Additionally, interferon regulatory factors (IRFs) exert critical control over macrophage phenotypic polarization, transition, and function. While IRF-1, IRF-5, and IRF-8 contribute to pro-inflammatory M1 formation, IRF-3 and IRF-4 govern M2 polarization (116). Furthermore, insulin-like growth factor 2 mRNA-binding protein 2 (IGF2BP2) mediates the conversion of M1 to M2 macrophages through an m6A-dependent mechanism targeting scleroderma-related protein 1 (117). Insulin has also been found to modulate macrophage polarization by activating the PI3K/Akt/Rac-1 and PPAR-γ signaling pathways (118). Cell surface receptor proteins also participate in M1 to M2 transition; for instance, dual blockade of TLR4 and TNFR1 promotes M1 to M2 polarization (119). MicroRNAs, like miR-126, play a pivotal role in shifting macrophages from pro-inflammatory M1 to anti-inflammatory M2 phenotype by downregulating VEGFA and KLF4 expression (120). In summary, macrophage polarization is intricately regulated by diverse factors, collectively shaping cellular function and phenotypic transitions.

### 2.5 M1/M2 macrophages phenotype correlates with cardiac rupture post-MI

Following MI, the heightened inflammatory response poses a significant risk of cardiac rupture (121, 122). Notably, clinical evidence underscores a direct correlation between the intensity of inflammation and the incidence of cardiac rupture in MI patients (123, 124). The risk of cardiac rupture post-MI is primarily associated with neutrophils, M1 macrophages, M2 macrophages, myeloperoxidase (MPO), and matrix metalloproteinases (MMPs), especially the infiltration of macrophages (125, 126). In the aftermath of MI, macrophages exhibit a biphasic activation pattern: pro-inflammatory M1 macrophages peak within the initial 3 days, while pro-fibrotic/repairing M2 macrophages reach their zenith around day 7 post-MI (127). Building upon this understanding, recent investigations have sought to elucidate the distinct roles played by different macrophage subtypes in modulating the risk of cardiac rupture. One study found that administration of the natural tetrapeptide Acetyl-Ser-Asp-Lys-Pro (Ac-SDKP) reduced the number of M1 macrophages in cardiac tissue post-MI, thereby significantly decreasing the incidence of cardiac rupture (128). Another study promoted apoptosis of M1 macrophages by knocking out apoptosis inhibitor of macrophage (AIM), consequently reducing the occurrence of cardiac rupture post-MI (129). Moreover, MMP-28 has garnered attention for its ability to augment the activation of M2 macrophages, thereby exerting a protective effect against cardiac rupture following MI (130).Furthermore, studies focusing on macrophage-specific Lgr4 deletion have unveiled a compelling mechanistic link, wherein ablation of this receptor culminates in a discernible shift in macrophage subtype composition within the infarcted milieu, characterized by a decrease in M1 macrophages juxtaposed with an augmentation in M2 macrophages, ultimately translating into a lowered incidence of cardiac rupture (65). Collectively, these findings underscore the pivotal role played by the delicate balance between M1 and M2 macrophages in dictating the susceptibility to cardiac rupture post-MI.

In summary, a comprehensive understanding of the intricate immunological landscape post-MI, with a specific focus on the nuanced modulation of macrophage subpopulations, holds immense promise in delineating novel therapeutic strategies aimed at mitigating the risk of cardiac rupture. These insights not only deepen our appreciation of the pathophysiological underpinnings of post-MI complications but also pave the way for the development of targeted interventions with translational potential, heralding a new dawn in the realm of cardiovascular medicine.

## 3 The role of different phenotypic macrophage-derived exosomes in IHD

Exosomes carry a cargo of proteins, RNA, DNA, lipids, and metabolites (such as amino acids, ATP, and acylamide) from the cell surface and interior. The types and levels of exosome cargo are influenced by donor cells, microenvironment, or physiological conditions. Through endocytosis, direct membrane fusion, or binding to cell surface receptors, exosomes can effectively target and deliver these biomolecules carrying important information to recipient cells, playing multifaceted roles in altering cell phenotypes, regulating gene expression, controlling the recruitment of inflammatory cells, etc., and participating in the pathogenesis and development of IHD (36, 131–133). Exosomes mediate signal exchanges between immune cells and cardiomyocytes. Studying the exosome cargo and their functions not only provides insight into the communication between cells in healthy and diseased states but also lays a foundation for the clinical application of exosomes. (**Table 3**).

**Table 3:** Effect of macrophage-derived exosomes on IHD.

Source of exosomes	Cargos	Effect	Target pathway
M1-macrophages	miR-29a (134)	Accelerate cardiac fibrosis and inhibit vascular neogenesis	–
M1-macrophages	miR-155 (135)	Promote cardiac fibrosis	Sevenless 1/SON
M1-macrophages	miR-155 (136)	Inhibit angiogenesis Myocardial repair	Sirt1/AMPKα2;RAC1-PAK2
M1-macrophages	miR-4532 (137)	Inhibit angiogenesis Myocardial repair	SP1/NF-κB P65
M1-macrophages	miR-25–3p (138)	Inhibit angiogenesis Myocardial repair	MALAT1/miR-25–3p/CDC42
M2-macrophages	miR-148a (139)	Anti-inflammatory	TXNIP and the TLR4/NF-κB/NLRP3
M2-macrophages	CircUbe3a (140)	Inhibition of fibrosis	miR-138–5p/Rhoc axis
M2-macrophages	miR-378a-3p (141)	Inhibition of cardiomyocyte pyroptosis	NLRP3/Caspase-1/GSDMD
M2-macrophages	miR-12715p (142)	Inhibition of cardiomyocyte apoptosis	TLR4/NF-κB/NLRP3

### 3.1 Role of M1 macrophage-derived exosomes in IHD

Previous studies have primarily focused on the role of M1 macrophage-derived exosomes in promoting inflammatory responses; in fact, it also plays an important crosstalk role in mediating between macrophages and cardiac cells. In response to hypoxia/reoxygenation stimulation, miR-29a in exosomes secreted by activated M1 macrophages mediated cardiomyocyte pyroptosis (134). Through paracrine effects, M1 macrophage-secreted exosomes are well-targeted to CFs and ECs to accelerate cardiac fibrosis and inhibit vascular neogenesis. Exosomes derived from M1 macrophages exhibit high expression of miRNA-155, which acts as a paracrine regulatory factor for CF proliferation and inflammation. Through exosome-mediated targeting of CFs, miRNA-155 can down-regulate the expression of Son of Sevenless gene (Sos1) to inhibit fibroblast proliferation, and decrease the expression of the anti-inflammatory gene Suppressor of Cytokine Signaling 1 (Socs1) to accelerate the inflammatory response of CFs (135). As a typical multifunctional miRNA, miRNA-155 can simultaneously target multiple molecular nodes. When transferred to ECs through exosomes derived from M1 macrophages, miRNA-155 can inhibit the Sirtuin 1 (Sirt1)/protein kinase AMP-activated catalytic subunit alpha 2 (AMPKα2)-endothelial nitric oxide synthase and Rac family small GTPase 1 (RAC1)-p21 (RAC1)-activated kinase 2 (PAK2) signaling pathways, thereby reducing the angiogenic capacity of ECs and impairing cardiac healing (136). M1 macrophage-derived exosomes can also exacerbate ECs injury by targeting the transport of miR-4532 and activating the SP1 and NF-κB P65 signaling pathways (137). Additionally, the highly expressed lncRNA Metastasis-Associated Lung Adenocarcinoma Transcript 1 (MALAT1) in exosomes secreted by M1 macrophages can competitively bind with miR-25–3p in ECs, promoting the expression of Cell Division Cycle Protein 42 (CDC42), which in turn activates the Mitogen-Activated Protein Kinase (MEK)/Extracellular Signal-Regulated Kinases (ERK) pathway and inhibits angiogenesis and myocardial regeneration (138).

### 3.2 Role of M2 macrophage-derived exosomes in IHD

M2 macrophage-derived exosomes account for an essential part of the therapeutic role played by M2 macrophages. They carry miR-148a that inhibits the expression of thioredoxin-interacting protein (TXNIP) and inactivates the TLR4/NF-κB/NLRP3 inflammasome signaling pathway, thus playing a cardioprotective role (139). They also mediate CF proliferation, migration, and MF transformation by transferring CircUbe3a to recipient cells targeting the miR-138–5p/Rhoc axis (140). Concurrently, exosomes derived from M2 macrophages can regulate the death program of cardiomyocytes through various mechanisms, with numerous miRNAs playing a role. Concurrently, exosomes derived from M2 macrophages can regulate the death program of cardiomyocytes through various mechanisms, with numerous miRNAs playing a role. Specifically, miR-1271–5p reduces the apoptosis of cardiomyocytes by downregulating the expression of SOX6, whereas miR-378a-3p mitigates cardiomyocyte pyroptosis by inhibiting the expression of ELAVL1, thus destabilizing the NLRP3 inflammasome and subsequently blocking the activation of the NLRP3/Caspase-1/GSDMD pathway (141, 142). In summary, exosomes derived from M2 macrophages can mitigate the damage caused by IHD and improve the prognosis of the disease through various pathways, such as alleviating inflammatory injury, reducing cardiomyocyte death, and promoting cardiac repair. Therefore, they hold promise as potential sources for therapeutic exosomes.

## 4 The therapeutic potential of macrophages and their exosomes in IHD

Macrophages and their exosomes play important roles in the progression of IHD at different stages. M1 macrophages primarily mediate early inflammatory responses, while M2 macrophages regulate the cardiac repair process. As crucial mediators of intercellular communication, exosomes have significant roles in modulating immune responses and facilitating communication between macrophages, cardiac cells, and the microenvironment. Further exploration of the functions and specific mechanisms of macrophages and their exosomes holds promise for uncovering their potential therapeutic applications.

In recent years, regenerative medicine has been widely applied in the treatment of IHD. The efficacy of stem cell therapy, particularly mesenchymal stem cell (MSC) therapy, has been confirmed by numerous preclinical and clinical studies (143, 144). There is also abundant preclinical evidence supporting the use of exosome therapy in animal models (145). Macrophages, due to their specific functional roles in the pathological process of IHD, often serve as target cells in related research. For instance, the studies conducted by Deng et al. and Xu et al. explored the therapeutic mechanisms of MSC-derived exosomes in the treatment of MI, although they act through different signaling pathways, both studies demonstrated that they improved cardiac injury by modulating macrophage phenotypic polarization (146, 147). There have also been studies on directly transplanting macrophages for the treatment of IHD. As mentioned earlier, transplantation of M2 macrophages has shown beneficial effects in reducing cardiac fibrosis and improving heart function in both I/R rats and heart failure mice models (88, 112). Macrophages have the advantage of an innate ability to migrate and settle into damaged tissue, which contributes to the functional implantation of transplanted cells in the damaged heart (78). However, cell therapy itself faces challenges such as poor recruitment and survival rates after transplantation into ischemic hearts. Additionally, macrophage transplantation presents difficulties in maintaining activated macrophages and has lower clinical feasibility (148). Pretreatment of macrophages may be able to partially address these issues. Chen et al. pre-treated bone marrow-derived macrophages with a sodium-dependent glucose transporter 2 inhibitor (SGLT2i) before transplantation into a mouse model of MI. This resulted in the suppression of inflammation, reduction of myocardial cell apoptosis, and promotion of the transformation of native cardiac macrophages into the M2 phenotype, which contributed to the reduction of adverse ventricular remodeling after MI (149). Similarly, Podaru et al. stimulated bone marrow-derived monocytes with macrophage colony-stimulating factor (M-CSF) and IL-4 to induce their differentiation into M2 macrophages before transplantation, resulting in significant improvement in cardiac function and structure in MI mice. The highlight of this study is that the generated M2 macrophages not only enhance the reparative secretion profile of endogenous reparative macrophages but also possess good stability, maintaining an M2-like phenotype even in the inflammatory environment after MI (150). Overall, macrophage transplantation for the treatment of IHD has accumulated some preclinical research data. However, further optimization of cell delivery routes and studies involving the use of human cells are needed before entering clinical trials. In comparison, a more convenient alternative may be the direct delivery of exosomes that carry the therapeutic functions of parent cells. For macrophages, blocking the transport of M1 macrophage-derived exosomes and promoting targeted delivery of M2 macrophage-derived exosomes, or promoting macrophage transformation from the M1 to M2 phenotype through the uptake of exogenous exosomes can all contribute to the repair of damaged myocardial tissue.

Researchers have further developed and optimized exosomes based on their characteristics. In terms of the therapeutic benefits of exosomes themselves, although exosomes exhibit good targeting ability, they have low persistence, and the use of biologics that can prolong their duration of action would enhance their efficacy. Biomaterials, such as hydrogels, have been widely employed in the delivery of exosomes due to their excellent biocompatibility, stability, and mechanical properties, which effectively extend the duration of action and even enhance therapeutic effects (151). For instance, Zou et al. constructed a composite system called Gel@Exo by combining conductive hydrogel with umbilical cord MSC-derived exosomes to improve their therapeutic effects on MI. This composite system offers advantages such as controllable gel kinetics, injectability, conductivity matching with natural myocardium, adaptability to heartbeats, softness, dynamic stability, and good cellular compatibility. It significantly improves the retention time of exosomes in the heart and optimizes their therapeutic effects (152). Furthermore, engineering modifications of exosomes can enhance their stability, bioactivity, and target binding capability at both cellular and tissue-specific levels, thereby further improving their therapeutic efficacy for diseases (153, 154). Techniques such as pre-treating parent cells and incorporating self-assembling peptides into the exosomes membrane allow the generated exosomes to better cope with the complex physiological environment of ischemic hearts (155, 156).

## 5 M1/M2 macrophages and their exosomes in clinical application

Clinical trials of macrophages and their exosomes for the treatment of cardiovascular diseases are still in the early stages, but preliminary results have shown promise. In 2013, Perin et al. (NCT00824005) significantly improved the left ventricular ejection fraction (LVEF) of patients with chronic ischemic heart disease by administering non-expanded autologous bone marrow macrophages via transendocardial injections. This trial demonstrated the potential positive outcomes of ex vivo expansion of macrophages for cardiac repair in patients with chronic ischemic heart disease (157). Further investigations revealed a total of 11 completed clinical studies on cell therapy using macrophages, focusing on conditions such as cardiomyopathy, arterial diseases, tumors, and other ailments. Among them, three trials (NCT01670981, NCT01020968, and NCT00765518) involved intramyocardial injection of Ixmyelocel-T, which contained a mixture of macrophages, granulocytes, monocytes, mixed myeloid progenitor cells, lymphocytes, and mesenchymal stem/stromal cells, to treat heart failure due to ischemic dilated cardiomyopathy. These studies showed a reduction in major adverse cardiovascular events and improvement in symptoms, attributing the effectiveness to the M2 macrophages in Ixmyelocel-T. These M2 macrophages were found to be effective in removing apoptotic cells, limiting tissue damage, and promoting wound healing, confirming the efficacy of macrophage therapy (158, 159).Additionally, two studies on autologous M2 macrophage therapy in children with severe cerebral palsy and patients with non-acute stroke confirmed the clinical efficacy of M2 macrophages in promoting neurological recovery without serious adverse events or cellular rejection (160, 161), further demonstrating the safety and efficacy of this approach.

As for exosome-related clinical trials, research has focused on exosomes secreted by plasma, mesenchymal stem cells, and human induced pluripotent stem cells for conditions like cancer, myocardial infarction, and decompensated cirrhosis. However, there have been no studies on exosomes secreted by M1 and M2 macrophages for the treatment of ischemic heart disease (IHD) yet. Nonetheless, a clinical trial investigating the phenotype of macrophages and their exosomes in type 2 diabetic patients who develop myocardial infarction (NCT02768935) suggested that the treatment of IHD with macrophages and their exosomes was progressing toward clinical therapy.

Overall, while there are fewer clinical trials involving macrophages and their exosomes in IHD, the available data have laid the initial groundwork for the development of new therapeutic strategies. As more studies are conducted, we can expect to gather more clinical evidence regarding the potential use of macrophages and their exosomes in the treatment of cardiovascular diseases.

## 6 Perspective

From the perspective of the delivery characteristics of exosomes, as endogenous extracellular vesicles, they possess excellent blood stability, substance transport properties, and high targeting ability, making them promising candidates as nanocarriers for drug delivery (162–165). This has the potential to overcome the limitations associated with traditional treatments for CVDs, such as low bioavailability, poor retention, inadequate targeting, and complex drug resistance. By utilizing methods such as ultrasound, transfection, incubation, transgenesis, freeze-thaw cycles, and heat shock, drugs can be loaded into exosomes for delivery, aiming to achieve therapeutic effects and improve prognosis (162). The study by Gao et al. utilized macrophage-derived exosomes as a nanoplatform for loading the anti-inflammatory drug methylprednisolone acetate, enhancing its anti-inflammatory and antioxidant effects, rescuing the viability of an *in vitro* inflammatory cardiomyocyte model, and providing preliminary evidence for the effectiveness of macrophage-derived exosomes as a drug carrier for cardiac immunotherapy (166). On the other hand, due to their abundant cell sources and diverse cargo, exosomes exhibit complex functional heterogeneity, which presents tremendous potential in clinical applications, including disease diagnosis. For instance, several studies have indicated that non-coding RNAs, such as miR-183, miR-21, and miR-208a, found in circulating extracellular vesicles, are closely associated with myocardial ischemic injury and hold promise as future biomarkers for IHD (167–169).

## 7 Conclusion

In summary, macrophages and their exosomes actively participate in and regulate the progression of IHD, potentially serving as key therapeutic tools for IHD treatment in the future. Additionally, ongoing optimization of cell therapy and advanced engineering techniques for exosomes are driving the development of related therapeutic strategies. Further exploration of the functions and mechanisms of macrophages and their exosomes will contribute to a deeper understanding of the pathophysiological mechanisms underlying IHD and provide new insights for its treatment.

## Author contributions

MW: Visualization, Writing – original draft, Writing – review & editing. CL: Writing – original draft, Writing – review & editing. YL: Writing – original draft, Writing – review & editing. YJ: Visualization, Writing – review & editing. YY: Writing – review & editing. XT: Writing – review & editing. CZ: Writing – review & editing.
